# Supplementary material for: Identification of photoperiod-induced specific miRNAs in the adrenal glands of Sunite sheep (Ovis aries)
Source: Front Vet Sci. 2022 Jul 22;9:888207. doi: 10.3389/fvets.2022.888207 (PMC9354845; doi:10.3389/fvets.2022.888207)
Supplement: Supplementary file 1 [file Data_Sheet_1.ZIP › Supplementary file/miRDeep2 file.zip!/miRDeep2 file/Know_Structure/LP42A3/oar-mir-410.pdf]

miRBase precursor : oar-mir-410
Total read count : 1991
oar-miR-410-5p read count : 4
oar-miR-410-3p read count : 1984
remaining reads : 3

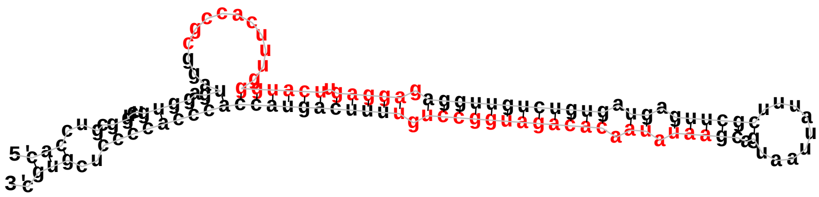

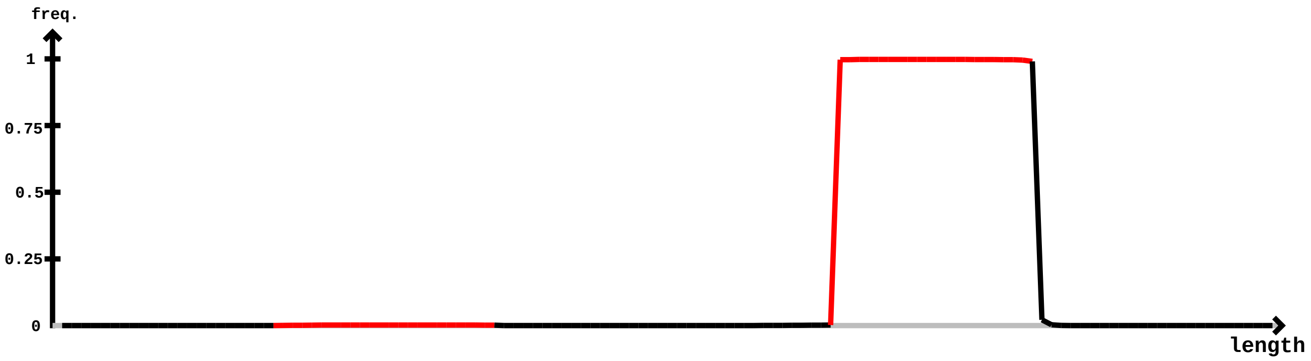


```
                                       oar-miR-410-5p
                                                                                           oar-miR-410-3p
5'- caccugcggucuguggaguaggcgccacuuuggguacuugaggagagguugucugugaugaguucgcuuuauuaaugacgaauauaacacagauggccuguuuucaguaccacccaccccucgugc  -3'   exp
    (((..(.(((..((((........))))....((((((.(((((.((((((((((((.((.((((((.........).))))).)).)))))))))))).))))))))))....))).)..))).   reads    mm
    ......................cgccacuuuggguacuugaggag.................................................................................   1        0
    .......................gccacuuuggguacuugaggag.................................................................................   1        0
    .........................cacuuuggguacuugagU...................................................................................   1        1
    ..........................acuuuggguacuugaggag.................................................................................   1        0
    ........................................................................uaaugacgaauauaacacagau................................   1        0
    ...........................................................................ugacgaauauaacacagauggc.............................   1        0
    .............................................................................acgaauauaacacagauggccugu.........................   1        0
    ...............................................................................gaauauaacacagauggccugu.........................   1        0
    ................................................................................aauauaacacagauggccu...........................   3        0
    ................................................................................aauauaacacagauggccug..........................   10       0
    ................................................................................aauauaacacagauggccugu.........................   1927     0
    ................................................................................aauauaacacagauggccuguu........................   37       0
    ................................................................................aauauaacacagauggccuguuu.......................   4        0
    ..................................................................................uauaacacagauggccuguuu.......................   1        0
    ..................................................................................uauaacacagauggccuguuuu......................   1        0
```
